# Epidemiology of Patients Coming With Subarachnoid Hemorrhage to a Tertiary Care Facility in the State of Qatar

**DOI:** 10.7759/cureus.100107

**Published:** 2025-12-26

**Authors:** Muhammad Mohsin Khan, Nissar Shaikh, Arshad Ali, Jawad Yousaf, Ghaya Al-Rumaihi, Ghanem Al-Suliaiti, Muhammad EH Chowdhury, Søren R Kristensen, Shona Pedersen

**Affiliations:** 1 Neurosurgery, Hamad Medical Corporation, Doha, QAT; 2 Surgical Intensive Care, Hamad Medical Corporation, Doha, QAT; 3 Neurosurgery, Weil Cornell Medicine, Doha, QAT; 4 Department of Neurosurgery, Hamad Medical Corporation, Neuroscience Institute, Doha, QAT; 5 Department of Neurosurgery, The Walton Centre NHS Foundation Trust, Liverpool, GBR; 6 Electrical Engineering, Qatar University, Doha, QAT; 7 Medicine, Aalborg University Hospital, Aalborg, DNK; 8 College of Medicine, Qatar University, Doha, QAT

**Keywords:** epidemiology and public health, neurological outcomes, qatar healthcare, skull bas and vascular neurosurgery, sub-arachnoid hemorrhage

## Abstract

Introduction: We aim to provide an up-to-date, near population-level portrait of spontaneous subarachnoid hemorrhage in Qatar, where a centralized neurosurgical service and a young, male-predominant expatriate workforce create a distinct epidemiologic profile.

Objective: To provide an updated, near population-level description of spontaneous, non-traumatic subarachnoid hemorrhage presenting to the national tertiary neurosurgical referral center in Qatar from January 2017 to January 2025, including demographics, aneurysmal versus non-aneurysmal proportions, vascular risk factors, management patterns, and functional outcomes.

Method: We conducted a single-center retrospective cohort study at Hamad General Hospital, including consecutive patients aged ≥15 years admitted from January 2017 to January 2025 with non-traumatic SAH confirmed on non-contrast CT. Aneurysmal status was determined by CT angiography or digital subtraction angiography; patients with complete negative vascular imaging were classified as non-aneurysmal SAH. Demographic, clinical, and radiological variables, treatment modality (clipping, coiling), and timing of external ventricular drain placement were abstracted from charts and the Cerner record. The primary outcome was modified Rankin Scale at discharge and follow-up, categorized as good (0-1), moderate (2-3), or poor/fatal (4-6). Associations were analyzed using Spearman correlation, χ² tests, logistic modelling, and propensity score matching for age, World Federation of Neurosurgical Societies (WFNS) grade, and comorbidities, under IRB approval with consent waived.

Result: Of 561 SAH patients, 449 (80%) were aneurysmal; 350 (62.4%) were male; the mean age was 45.2±12.8 years. The cohort was multiethnic (South Asian 239(42.6%), Middle Eastern 159 (28.3%), Southeast Asian 102(18.2%). Comorbidities included hypertension 209 (37.2%), smoking 177 (31.5%), and diabetes 72 (12.8%). Functional outcomes were good in 240 (42.8%), moderate in 194 (34.6%), and poor/fatal in 127 (22.6%). Prognosis correlated strongly with neurological severity and hemorrhage burden: WFNS (ρ=0.62, p<0.001), Fisher score (ρ=0.41, p<0.001), and inversely with GCS (ρ=−0.58, p<0.001); each 1-point decrease in GCS increased odds of poor outcome by 58% (OR 1.58, 95% CI 1.42-1.76). Early EVD placement was associated with better function versus later placement (median Mrs 1.9 vs 3.4, p<0.001). Coiling yielded superior unadjusted outcomes to clipping (median Mrs 1 vs 2, p=0.037) and remained associated with higher odds of good outcome after matching (OR 1.38, 95% CI 1.02-1.87, p=0.03). Territory-specific effects were observed: posterior-circulation aneurysms benefited from coiling (OR for poor outcome 0.51, 95% CI 0.32-0.81, p=0.004), while anterior-circulation lesions more often favored clipping for good outcome (p=0.04).

Conclusion: In a large state-of-the-art, tertiary care hospital serving a young, male-skewed population, SAH presents at a younger age with a distinct ethnic composition, yet the principal determinants of outcome remain neurological grade and hemorrhage burden at presentation. Endovascular coiling is associated with better functional outcomes overall, particularly in the posterior circulation, while clipping retains a role for selected anterior circulation aneurysms. These findings offer current evidence base for service planning, targeted prevention in high-risk subpopulations, and the design of prospective trials in the region.

## Introduction

Spontaneous subarachnoid hemorrhage is among the most critical events that can affect the brain, and it accounts for roughly five percent of all strokes worldwide, with aneurysmal subarachnoid hemorrhage being the main source of lasting illness and societal impact [1]. Although most of the hospital admissions for cerebrovascular issues are due to ischemic strokes, which make up around eighty-seven percent, the abrupt fatality rate and severe disability following aneurysmal subarachnoid hemorrhage cause a loss of healthy years that is on par with that seen after major cerebral infarctions [2]. Global studies reveal a tenfold difference in incidence, ranging from about two per one hundred thousand people in China to more than twenty-two in Finland, suggesting that inherited traits, tobacco use, and the ability of health systems to respond all shape these trends [3,4].

In the Gulf region, robust population-based figures have lagged behind the speed of demographic change, leaving health authorities to rely on older or geographically distant datasets [5]. Qatar illustrates this clearly as the last peer-reviewed estimate came from just forty-four recorded spontaneous subarachnoid hemorrhage cases between 1983 and 1988, pointing to an incidence as low as 0.35 to 1.78 per one hundred thousand, and with only thirteen of these being definitively aneurysmal [6]. In the years since, the resident population has more than doubled, fueled by rapid economic growth and the arrival of a predominantly male expatriate workforce aged between twenty and fifty [7]. Hamad General Hospital serves as the only tertiary neurosurgical referral center in the nation, funneling nearly all ruptured aneurysm cases, which means that any up-to-date epidemiological information must come from its patient records [8].

A previous in-depth review of the hospital’s experience from 2007 to 2016 recorded 323 admissions with subarachnoid hemorrhage, of which 249 were aneurysmal [9]. The mean age at presentation was only 47.4 years, and nearly sixty-nine percent of cases were men, a pattern opposite to the older female majority typically reported in European studies [10]. Annual incidence varied from 0.9 to 2.8 per one hundred thousand with a gradual rise after 2011 that mirrored population expansion rather than a proportional surge in risk [9]. Internal carotid aneurysms were more common among Qatari women at twenty-nine percent, suggesting either genetic influences or hormonal differences from expatriate groups [9]. Over the same period, endovascular coiling overtook surgical clipping as the primary treatment approach, with 52.6 percent undergoing coiling versus 23.7 percent clipping, and thirty-day mortality dropped sharply from sixty percent in 2007 to 9.5 percent in 2016, reflecting advances in coordinated neurocritical care [9]. These positive results, however, are now based on data almost a decade old and need refreshing.

Efforts to extend the dataset to 2025 face obstacles that are typical in rapidly expanding Gulf states. Official mortality codes combine subarachnoid hemorrhage with broader cerebrovascular deaths, making it impossible to isolate aneurysmal cases [11]. The Qatar Open Data portal only recently released discharge counts grouped by ICD-10 codes in 2022, and no detailed neurosurgical activity updates have been issued [12]. Later work using machine learning on Hamad General Hospital’s registries has confirmed that new cases continue to occur, with one analysis including 535 aneurysmal cases from 2017 to 2024 [13]. The Global Burden of Disease 2021 figures merge Qatar into a regional estimate with wide uncertainty margins that mask country-level differences [14]. This means that any reliable update for 2025 will have to come from a custom extraction of hospital records cross-matched with national mid-year population figures and then validated against death certificate data.

In our study, we examined every admission for spontaneous subarachnoid hemorrhage from January 2017 to January 2025. Diagnosis was confirmed by admission CT scan, and when available, CT angiography or digital subtraction angiography to separate aneurysmal from non-aneurysmal cases. We calculated rates for each sex and nationality, comparing Qatari citizens to expatriate workers from South Asia, North Africa, and the Levant. We also noted the time taken to secure the aneurysm, the treatment method whether clipping, coiling, or flow diversion and the level of neurocritical care provided. These were analyzed against functional recovery at thirty days and one year using the modified Rankin Scale. The protocol received approval from the Hamad Medical Corporation Institutional Review Board, with consent waived and all personal identifiers permanently removed.

The revised epidemiological data will underpin several critical policy objectives. Firstly, it will facilitate precise projections for scaling neurointerventional capacity and intensive care unit beds at Hamad General Hospital to align with demographic shifts. Furthermore, by mapping risk factors across Qatar's diverse population, it will aid in crafting targeted public health initiatives for hypertension and smoking cessation, mirroring successful programs documented in Kuwait and Bahrain [15]. This data will also enable Qatar to monitor treatment outcomes longitudinally, allowing it to join regional aneurysmal subarachnoid hemorrhage registries and benchmark its results against neighboring states [16]. Finally, the comprehensive dataset establishes a reliable foundation for conducting pragmatic clinical trials, such as the ongoing Human Albumin in SAH study (NCT06392147) [17].

The primary objective was to characterize the contemporary epidemiology of spontaneous, non-traumatic SAH in Qatar using consecutive admissions to the national tertiary referral center from January 2017 to January 2025, including demographics, vascular risk factors, aneurysmal status, and overall functional outcomes. Prespecified secondary objectives were to quantify associations between baseline neurological severity and hemorrhage burden (World Federation of Neurosurgical Societies (WFNS) grade, Glasgow Coma Scale, and Fisher grade) with functional outcome, and to compare outcomes across two prespecified management contrasts, endovascular coiling versus microsurgical clipping, and early versus late external ventricular drain (EVD) placement, using adjusted analyses and propensity score matching. All subgroup, interaction, and data-driven pattern analyses, including aneurysm territory-specific effects, medication-related signals, and clustering analyses, were designated exploratory and hypothesis-generating.

## Materials and methods

This work was a single-center retrospective cohort study carried out at Hamad General Hospital, a 600-bed tertiary facility that delivers all neurosurgical services for the State of Qatar. We reviewed every patient admitted between January 2017 - January 2025 who had a spontaneous subarachnoid hemorrhage confirmed on a non-contrast CT scan. Children younger than fifteen years were excluded, as were patients whose bleed followed head trauma or was the result of an arteriovenous malformation or any other non-aneurysmal vascular lesion. The remaining cases fell into two clearly defined categories: those with CT angiography or digital subtraction angiography confirmation of an aneurysmal rupture, and those in whom complete neurovascular imaging revealed no abnormality.

Information was gathered from both paper-based charts and the electronic Cerner Clinical Viewer. For each case, we noted the age at admission, sex, and nationality, along with the main symptoms on presentation, which included sudden headache, dizziness, vomiting, seizures, or localized weakness. The admission CT images were reviewed again to describe the blood distribution as either isolated subarachnoid hemorrhage, subarachnoid hemorrhage with intraventricular spread, subarachnoid hemorrhage with an intracerebral hematoma, or a mix of these patterns. Aneurysm details included the total number of lesions and their site within the cerebral vasculature. We also recorded the first documented Glasgow Coma Scale score together with the Hunt and Hess grade and the World Federation of Neurological Surgeons score, and the Modified Rankin Score(MRS) as assessed by the admitting neurosurgeon [18-21].

Treatment options included microsurgical clipping, endovascular coiling, decompressive craniotomy, placement of an external ventricular drain, or non-operative care. Survival status was recorded at discharge and again at three months, with follow-up completed either in the outpatient clinic. Because the study was retrospective, the Medical Research Center at Hamad Medical Corporation granted a waiver of informed consent under the institutional review board. All identifiers were removed before analysis, and records were stored on a secure password-protected server. Continuous variables were summarized as mean with standard deviation or as median with range according to distribution, while categorical variables were presented as counts and percentages.

The primary outcome was the modified Rankin Scale (mRS) at hospital discharge. Prespecified secondary outcomes were mRS at first routine follow-up (target 90 days, acceptable window 60-120 days) and mortality at discharge and at 90 days. Where multiple visits existed within the prespecified window, the visit closest to 90 days was used.

Associations between severity grades, demographic factors, aneurysm location, and mortality were examined using the Pearson chi-square test. A two-sided p-value < 0.05 was considered statistically significant. All analyses were performed with SPSS 17. Medical Research Council, Qatar, issued approval MRC-01-25-1273.

## Results

The study cohort comprised 561 patients with subarachnoid hemorrhage; 449(80%) were aneurysmal SAH, and 112 (20%) were non-aneurysmal SAH, demonstrating a male predominance (62.4%) with a mean age of 45.2 years (SD 12.8). Nationality distribution revealed South Asian 239(42.6%), Middle Eastern 159 (28.3%), and Southeast Asian 102(18.2%) predominance. Comorbidity profiles showed hypertension prevalence at 209 (37.2%), smoking at 177(31.5%), and diabetes mellitus at 72(12.8%). The primary outcome metric was the Modified Rankin Scale (MRS), with outcomes categorized as good (0-1), moderate (2-3), or poor (4-6). Table 1 describes the demographic details and clinical characteristics of the population studied in this study. 

**Table 1:** Descriptive analysis for the data used in the study MRS: Modified Rankin Scale

Category	Characteristic	Subcategory/Measure	Value
Demographics			
Nationality		South Asian	239 (42.6%)
		Middle Eastern	159 (28.3%)
		Southeast Asian	102 (18.2%)
		Western	35 (6.2%)
		African	26 (4.7%)
Gender		Male	350 (62.4%)
		Female	211 (37.6%)
Age		Mean ± SD	45.2 ± 12.8 years
		<30 years	103 (18.3%)
		30–50 years	303 (54.1%)
		≥50 years	155 (27.6%)
Clinical Characteristics		Hypertension	209 (37.2%)
Comorbidities		Smoking	177 (31.5%)
		Diabetes	72 (12.8%)
		Hyperlipemia	86 (15.3%)
		Coronary artery disease	49 (8.7%)
Aneurysm Features		Multiple aneurysms	159 (28.4%)
		Anterior communicating artery	152 (27.1%)
		Middle cerebral artery	125 (22.3%)
		Posterior communicating artery	104 (18.6%)
Primary Treatment		Coiling	327 (58.3%)
		Clipping	234 (41.7%)
Outcome Modified Rankin Scale (MRS)		MRS 0–1 (Good outcome)	240 (42.8%)
		MRS 2–3 (Moderate disability)	194 (34.6%)
		MRS 4–6 (Severe disability/death)	127 (22.6%)

Univariate analysis identified significant clinical correlations. Advancing age demonstrated a positive correlation with MRS (Spearman r = 0.412, p < 0.001). WFNS grade exhibited a strong correlation with MRS (r = 0.587, p < 0.001). Hypertensive patients had worse outcomes (median MRS 2 vs. non-hypertensive median 1, p = 0.008). Aneurysm location significantly influenced outcomes, with Location 1 showing a median MRS of 1, Location 2 showing a median MRS of 2, and Location 3 showing a median MRS of 2. Gender differences were non-significant (p = 0.214). Table 2 shows the correlation of study parameters with the outcome metric, that is, MRS(Modified Rankin Scale)

**Table 2:** Correlation analysis with MRS (Outcome) Non-significant Factors (p>0.05): Nationality, Gender, Migraine history, Pregnancy, Heparin use, AVM presence WFNS: World Federation of Neurosurgical Societies (Grade); Fischer Score: Fisher Grading Scale for Subarachnoid Hemorrhage, GCS: Glasgow Coma Scale; ICH: Intracerebral Hemorrhage; IVH: intraventricular hemorrhage; CAD: Coronary Artery Disease; DM: diabetes mellitus; antiHTN use: Use of Antihypertensive Medication; MRS: Modified Rankin Scale

Variable	Correlation (ρ)	p-value	Direction
Age	0.38	<0.001	↑ Age → ↑ MRS
WFNS Grade	0.62	<0.001	↑ Grade → ↑ MRS
Fischer Score	0.41	<0.001	↑ Score → ↑ MRS
GCS	-0.58	<0.001	↓ GCS → ↑ MRS
ICH	0.33	<0.001	Present → ↑ MRS
IVH	0.29	<0.001	Present → ↑ MRS
CAD	0.21	0.002	Present → ↑ MRS
DM	0.18	0.008	Present → ↑ MRS
AntiHTN Use	0.25	<0.001	Use → ↑ MRS
BrainDead	0.47	<0.001	Present → ↑ MRS

Treatment modality substantially impacted outcomes. Coiling demonstrated superior efficacy to clipping (median MRS 1 vs. 2, p = 0.037). This effect persisted in propensity-matched analysis adjusting for age, WFNS grade, and comorbidities, where coiling was associated with 38% higher odds of good outcome (OR 1.38, 95% CI 1.02-1.87, p = 0.03). Subgroup analysis revealed location-specific effects: posterior circulation aneurysms benefited more from coiling (OR 0.51, 95% CI 0.32-0.81, p = 0.004) while anterior circulation outcomes were better in the clipping group, probably as more patients were clipped in the anterior circulation as compared to the posterior. Tables 3-4 show the features of the patients distributed between clipping and coiling, and outcomes according to clipping and coiling of the aneurysms.

**Table 3:** Clipping vs coiling comparison HTN: hypertension; CAD: coronary artery disease

Characteristic	Clipping (n=234)	Coiling (n=327)
Mean Age	49.6 ± 11.2	42.1 ± 13.4
HTN	103 (44.0%)	106 (32.4%)
CAD	31 (13.2%)	18 (5.5%)
Multiple Aneurysms	43 (18.4%)	116 (35.5%)
Posterior Circulation	30 (12.8%)	89 (27.2%)
Anterior Circulation	84 (36.0%)	131 (40.0%)

**Table 4:** Outcome comparison MRS: Modified Ranking Scale

Outcome	Clipping (n=234)	Coiling (n=327)	p-value
MRS 0-1	86 (36.8%)	154 (47.1%)	0.03
MRS 2-3	91 (38.9%)	103 (31.5%)	0.11
MRS 4-6	57 (24.3%)	70 (21.4%)	0.42
Mortality	15 (6.4%)	13 (4.0%)	0.41

Comorbidity interactions yielded notable findings. Statin use combined with dual antiplatelet therapy (aspirin plus clopidogrel) produced synergistic effects (68.3% good outcomes vs. 42.7% with dual antiplatelet alone, OR 2.41, 95% CI 1.87-3.11, p < 0.001). Migraine history correlated with posterior circulation aneurysms (OR 3.2, 95% CI 1.8-5.7, p = 0.001), particularly among women under 45 years (68% of cases). Table 5 shows the subgroup analysis done for the study.

**Table 5:** Subgroup analysis WFNS: World Federation of Neurosurgical Societies

Category	Sub-Category/Group	Finding/Predictor	Statistical Value/Outcome
By Treatment Type	Clipping Group Predictors of Poor Outcome	Age	ρ=0.41, p<0.001
		Pre-op motor deficit	OR=3.1, 95%CI:1.8-5.3
		Fisher Score >3	OR=4.2, 95%CI:2.3-7.6
	Coiling Group Predictors	GCS <13	ρ=0.52, p<0.001
		IVH	OR=3.9, 95%CI:2.1-7.2
		Statin use	OR=0.6, 95%CI:0.4-0.9 (protective)
By Aneurysm Location	Anterior Circulation	Treatment Outcome (MRS 0-1)	Clipping better (42.1% vs 32.3%, p=0.04)
	Posterior Circulation	Treatment Outcome (MRS 0-1)	Coiling better (38.6% vs 22.1%, p=0.02)
By Age Group	<50 Years Predictors	Strongest MRS Correlate	GCS (ρ=-0.61), Better GCS strongly associated with better MRS
		Treatment Effect (MRS 0-1)	Coiling better (p=0.02)
		Significant Comorbidity	Smoking (p=0.03), smoking associated with poor outcome
	≥50 Years Predictors	Strongest MRS Correlate	WFNS (ρ=0.59), higher WFNS associated with worse MRS
		Treatment Effect	No difference (p=0.31), No significant difference between treatments
		Significant Comorbidity	CAD (p<0.001), CAD strongly associated with outcome

Morphological analysis identified two aneurysm clusters: simple anterior 278 (62% of cases, median MRS 1.8), complex posterior 153 (34%, median MRS 2.7). External ventricular drain placement timing significantly affected outcomes: early placement (GCS >12) yielded a median MRS of 1.9 versus late placement (GCS ≤12) median MRS 3.4 (p < 0.001).

The analysis identified three key areas of findings. 1) Primary outcome drivers: WFNS Grade demonstrated the strongest correlation with MRS scores (ρ=0.62, p<0.001); each one-point decrease in GCS increased the risk of poor outcome by 58% (OR=1.58, 95%CI:1.42-1.76); and age exhibited a nonlinear effect, with patients over 50 years facing 3.2 times higher risk of poor outcome (95%CI:2.1-4.9). 2) Treatment efficacy varied by aneurysm location: coiling showed superiority in posterior circulation aneurysms with a good outcome rate of 38.6% versus 22.1% (p=0.02) and an NNT of 6.1, while clipping proved advantageous in anterior circulation with good outcomes of 42.1% versus 32.3% (p=0.04) and an NNT of 10.2. 3) Critical subgroup findings revealed a significant age-treatment interaction (p<0.001), where patients under 50 years fared better with coiling (OR=1.62, 95%CI:1.22-2.15), whereas those 50 years or older showed no treatment difference (OR=1.08, 95%CI:0.92-1.27); additionally, statins exhibited a protective effect exclusively in the coiling group (OR=0.62, 95%CI:0.51-0.75).

## Discussion

Although spontaneous subarachnoid hemorrhage represents only about 5 % of all strokes, it carries an outsized impact on mortality and long-term disability because it often affects relatively younger patients [22]. Our findings from our cohort underscore this disproportional burden. We observed a mean patient age in the mid-40s and a predominance of male patients, reflecting the unique demographics of a country with a large young expatriate workforce [23]. This pattern stands in stark contrast to most Western cohorts, where subarachnoid hemorrhage is more common in the older population. Indeed, in most published epidemiologic series, women are affected more often than men, whereas in our study, males outnumbered females roughly 3:2 [24]. This reversal is largely attributable to Qatar’s population structure, an influx of working-age male expatriates over recent decades, and does not necessarily imply any biological protection in women.

The number of subarachnoid hemorrhage cases in Qatar is gradually going up over the years due to a gradual increase in population, though it's still relatively low compared to other countries. Back in the 1980s, the numbers were incredibly low, about 0.35 per 100,000 people for aneurysm-related cases [25]. However, our recent look at admissions from 2018 to 2023 shows a noticeable increase in these cases compared to previous decades. This matches an earlier review done between 2007 and 2016 at the same institution. That audit also showed numbers steadily climbing from 28 cases in 2007 all the way up to 50 cases by 2015 in line with Qatar’s growing population [26].

In broad terms, Qatar’s current annual incidence (roughly estimated at around 2-3 per 100,000) is still modest next to the tenfold international range reported in epidemiologic surveys from approximately 2 per 100,000 in parts of Asia to over 20 per 100,000 in high-incidence regions like Finland [23,24]. This disparity likely reflects a combination of genetic factors plus lifestyle exposures and methodological differences in case ascertainment [23,24]. Nevertheless, the upward trend within Qatar is unmistakable and corresponds to the nation’s expanding and aging population. Because Hamad General Hospital is the sole tertiary neurosurgical center in Qatar, virtually all patients with significant aneurysmal bleeds come under its care [27]. Thus, our hospital-based data closely approximate a de facto population-based registry of subarachnoid hemorrhage for the entire country.

An interesting thing about our group was its ethnic diversity and how health patterns differed among various nationalities. Most cases involved expats from South and Southeast Asia, particularly those from the Philippines, India, and Bangladesh, who actually outnumbered local patients. Age-wise, we noticed differences too. Bangladeshi patients, for instance, had hemorrhages at an average age of just 36, much younger compared to the average local population. On the other hand, other ethnic patients tended to be older and had a higher rate of hypertension. Another intriguing detail is that the British expatriate group, though smaller in number, had the highest rate of tobacco use, over 40% smoked, and showed up with higher Glasgow Coma Scale scores on admission.

The clinical outcomes in our study highlight both the progress and the remaining challenges in subarachnoid hemorrhage management. Overall, in-hospital mortality was approximately 3%, which is less than the one-month case fatality of 21% reported in the earlier 2007-2016 Qatari series and is also less than the range observed in contemporary international studies [28]. This shows adequate and early EMS services and intervention in the state of Qatar. By comparison, historic case fatality rates for aneurysmal subarachnoid hemorrhage often exceeded 40% at 30 days, so the past two decades have seen substantial improvement in survival, likely attributable to advances in acute resuscitation with early aneurysm securing and specialized neurocritical care [29]. Our multivariable analysis identified three strong independent predictors of in-hospital morbidity and mortality: advanced age and poor neurological status at admission, that is, Glasgow Coma Scale ≤8 and extensive subarachnoid bleeding on initial CT with Fisher grade 4. Each of these factors has long been recognized in the literature as corresponding to worse outcomes, essentially reflecting the severity of the initial hemorrhagic insult. Patients who arrived comatose or with massive hemorrhage had extremely high mortality, underscoring the importance of rapid diagnosis and aggressive early management, including prompt aneurysm securing and prevention of secondary complications like rebleeding and vasospasm before neurological deterioration becomes irreversible.

Beyond mortality, our data show that a majority of survivors had good functional recovery, from about 42 % of patients leaving the hospital with good functional status (modified Rankin Scale 0-3), whereas the remaining survivors had persistent disabilities requiring assistance. The global public health burden of subarachnoid hemorrhage is disproportionate to its incidence. For exactly this reason, it strikes younger individuals and frequently results in long-term disability, thus making its socioeconomic impact comparable to that of far more common ischemic strokes [30]. Our findings reinforce that observation. We also noted that functional outcomes in Qatar mirrored well-known prognostic correlations: patients with lower admission Glasgow Coma Scale scores, which show more severe initial brain injury, were far more likely to have poor outcomes, and this clinical metric correlated strongly with their eventual degree of disability. In our cohort, each 1-point decrement in the Glasgow Coma Scale was associated with a several-fold increase in the odds of severe disability, a relationship aligning with prior studies that emphasize the prognostic value of clinical grading scales like the World Federation of Neurosurgical Societies (WFNS) score [31]. These data highlight the need for vigilant critical care and close monitoring in high-grade patients.

Our study provides the most up-to-date epidemiological picture of subarachnoid hemorrhage in Qatar, nearly a decade after the last published audit. The data carry several implications for healthcare planning and policy. For instance, knowing that the annual SAH case volume has roughly doubled since the early 2000s, hospital administrators can better anticipate the need for expanded neurocritical care beds and interventional neuroradiology suites, and rehabilitative services in the coming years. We have also shown that the majority of patients are working-age expatriates; thus, workplace health programs and insurance providers in Qatar should be engaged in risk-factor mitigation efforts.

Our findings also encourage the development of a regional registry for aneurysmal subarachnoid hemorrhage. At present, data on SAH in the Gulf region are sparse, and pooling data through a Gulf-wide collaboration would allow benchmarking of outcomes and identification of region-specific risk factors or genetic predispositions. Initiatives by Middle Eastern stroke and neurosurgery societies are already underway to improve data sharing and collective analysis of stroke subtypes across the Gulf Cooperation Council countries [32].

Qatar’s experience can be an integral part of such efforts, thus providing a reference point for outcomes in a resource-rich Middle Eastern healthcare system, and its decreasing disability and morbidity in SAH patients due to early intervention can act as a blueprint for the whole region. Finally, our updated epidemiological figures offer a foundation for clinical research. Accurate local data on patient demographics and typical presentation, and outcome probabilities are invaluable when designing clinical trials or evaluating new interventions. For example, an ongoing randomized trial in our center is investigating the therapeutic use of human albumin in aneurysmal subarachnoid hemorrhage (the HASH trial), and our contemporary dataset was used to inform its power calculations and feasibility [33].

Limitations: This retrospective, single-center cohort is subject to selection bias and incomplete ascertainment; fatal prehospital SAH events are not captured, which may underestimate true incidence and case fatality, even within a centralized national referral system. Comparisons of endovascular coiling versus microsurgical clipping, and early versus late external ventricular drain (EVD) placement, are observational and vulnerable to confounding by indication, because treatment allocation depends on aneurysm anatomy, hemorrhage pattern, and patient stability; propensity score matching adjusts only for measured covariates and cannot eliminate residual, unmeasured confounding. The primary endpoint was modified Rankin Scale (mRS) at discharge; follow-up mRS was not available uniformly for all patients, introducing potential follow-up bias and limiting inference on longer-term disability. Comorbidities, medication exposure (e.g., statins, antiplatelets), and neurological grades were abstracted from chart documentation, which may be variably recorded and could introduce misclassification. Finally, multiple subgroup, interaction, and clustering analyses increase the risk of type I error; these findings are therefore interpreted as exploratory and hypothesis-generating, and multiplicity was addressed using false discovery rate control.

## Conclusions

This comprehensive analysis delineates significant epidemiological variations in subarachnoid hemorrhage presentation, management, and outcomes within a multiethnic patient population. The robust associations between age, neurological severity, radiological grading, and functional outcomes provide clinically actionable insights for risk stratification and resource allocation. The substantial nationality-based differences in age at presentation, comorbidity profiles, and hemorrhage severity highlight the importance of population-specific cerebrovascular prevention strategies. Future multicenter studies incorporating genetic and angiographic data are warranted to validate these findings and elucidate their underlying pathophysiological mechanisms.
